# YAP Attenuates CD8 T Cell-Mediated Anti-tumor Response

**DOI:** 10.3389/fimmu.2020.00580

**Published:** 2020-04-08

**Authors:** Andriana Lebid, Liam Chung, Drew M. Pardoll, Fan Pan

**Affiliations:** Immunology and Hematopoiesis Division, Department of Oncology, Bloomberg-Kimmel Institute, Sidney Kimmel Comprehensive Cancer Center, Johns Hopkins University School of Medicine, Baltimore, MA, United States

**Keywords:** CD8 T cell, YAP, tumor, immunesuppression, hippo

## Abstract

YAP is a transcriptional coactivator of the Hippo signaling pathway that has largely been studied for its role in the regulation of organ size during development. Several studies have shown that YAP is upregulated in cancer cells, and more recently in the T regulatory (Treg) subset of CD4+ cells. These observations suggest that the transcriptional co-activator may promote tumor persistence and progression. Here, we report that YAP also plays an immunoinhibitory role in CD8 T cells, especially in activated cytotoxic cells usually found in the tumor microenvironment. Our findings add further rationale for the development and use of pharmacologic inhibitors of YAP to treat cancer.

## Introduction

The Hippo signaling pathway was originally discovered for its control of cell proliferation in *Drosophila melanogaster* (1, 2). This name arose because tissue-specific overexpression of the key Hippo transcription factors or deletion of upstream Hippo pathway kinases caused overgrowth of organs such as the liver and the intestine (3, 4). Since then, many more roles of this pathway in mammals have been discovered, including cell survival, proliferation, stemness, and regeneration (5).

The major well-studied downstream players of the Hippo pathway are mammalian sterile 20-like kinase 1 and 2 (MST1/2), large tumor suppressor 1 and 2 (LATS1/2), transcription coactivator with PDZ-binding motif (TAZ), and Yes-associated protein (YAP) (5). YAP is a transcriptional coactivator that has largely been studied for its role in the regulation of organ size during development. Normally, the transcriptional co-activators YAP and TAZ are degraded in the cell cytoplasm by the adaptor 14-3-3 through phosphorylation-dependent degradation that is controlled by the LATS1/2 kinase, which is activated upon phosphorylation by MST1/2 (6). When the Hippo pathway is deactivated, YAP and TAZ fail to be degraded, and traffic into the nucleus where they can alter gene expression by interacting with transcription factor TEA domains (TEADs) (7).

An abundance of literature already links dysregulation of the Hippo signaling pathway to cancer progression (5, 8–12). Generally, YAP and TAZ are thought of as oncogenes whose hyperactivity enhances cell survival and proliferation of tumor cells. However, as more is becoming known about the Hippo pathway, non-canonical roles of its components are being discovered in immune cells that play a role in the tumor progression (13–17). The tumor environment has many hallmarks that include genome instability, angiogenesis, replicative immortality, and evasion of destruction by the immune system. Whereas abnormal cells are usually eliminated by the immune system, the immunosuppressive cancer sites undergo immunoediting until they can escape elimination. Current cancer immunotherapy goals include reprogramming of myeloid-derived suppressor cells (MDSCs), reactivation or expansion of cytotoxic CD8 cells, and inactivation or reduction of suppressive T regulatory (Treg) cells. Studies have shown that YAP promotes growth and proliferation of cancer cells, and more recently that it also enhances differentiation of the immunosuppressive T regulatory (Treg) subset of CD4+ cells (5, 8, 10, 12–14). Here, we report that YAP also plays an immunoinhibitory role in CD8 T cells, especially activated cytotoxic cells usually found in the tumor microenvironment. Given mounting evidence about the efficacy of the Hippo pathway small molecule inhibitors in cancer, it is key to understand how these drugs may also affect the tumor immune microenvironment, especially CD8 cells (18–21).

## Results

### YAP Is Expressed in Activated CD8 Cells

**Figure 1:**
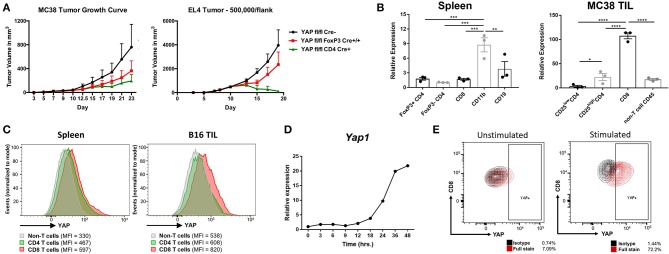
YAP is expressed in activated CD8 T cells. **(A)** Tumor growth kinetics of WT vs YAP-deficient animals challenged with MC38 or EL4 (*s.c*.) cells are depicted. **(B)** *Yap1* mRNA expression in immune cell subsets sorted from healthy spleens or MC38 tumors was quantified using RT-qPCR. **(C)** YAP expression was detected by intracellular flow cytometry in the B16 tumor-bearing mouse spleen vs tumor. **(D)** Kinetics of *Yap1* mRNA expression during OTI CD8 cell activation with SIINFEKL peptide and IL2 were quantified using RT-qPCR. **(E)** YAP protein expression was detected by intracellular flow cytometry in unstimulated vs activated OTI CD8 cells (24 h). Data information: **(A,B)** represent mean ± SEM. In (**B)**, *P < 0.05; ^**^P < 0.01; ***P < 0.001; ****P < 0.0001 by a two-way ANOVA test. *N* = 5–8/group in **(A)**. Results in **(A,C,D)** are representative of two independent experiments.

We discovered that YAP plays a role in non-Treg T cells through tumor studies of the YAP fl/fl CD4 Cre and YAP FoxP3 YFP Cre animals (T cell and Treg-specific deletion of YAP). The YAP CD4 Cre mice always had a more dramatic anti-tumor phenotype across several subcutaneous murine tumor models, including MC38 and EL4 (Figure 1A). This indicated that YAP had another immune-inhibitory role in non-Treg cells - conventional CD4 and/or CD8 cells. The CD4 Cre model deletes floxed genes in both CD4+ and CD8+ cells during the double-positive stage of development, albeit less thoroughly in CD8 T cells than the CD4 cells because the Cre recombinase is transiently—rather than constitutively—expressed in CD8s compared to the CD4s (22).

To observe which immune cells expressed YAP, major immune cell types from naïve spleens vs. MC38 tumors of C57BL/6 mice were sorted. While the CD11b+ subset from the spleens of healthy mice expressed most YAP, the MC38 CD8s TlLs (tumor-infiltrating lymphocytes) expressed most YAP message compared to other immune cell subsets (Figure 1B, Figure S1). Consistent with our group's previous findings, the FoxP3+ subset of CD4 cells expressed more YAP than FoxP3- CD4s in both settings (13). Intracellular flow cytometry staining in tumor-draining lymph nodes vs B16F10 tumor-infiltrating lymphocytes (TILs) revealed that intratumoral CD8s had upregulated YAP protein expression (Figure 1C). Tumor-infiltrating CD8s are largely activated, expressing cytotoxic effectors such as Perforin and Granzyme B (including plentiful IFNγ), which are scarce in murine CD8s located in lymphoid organs at homeostasis (23). A greater frequency of these cells also have upregulated exhaustion markers such as CTLA-4, GITR, KLRG1, Lag-3, PD-1, and Tim-3 (Figure S2). Thus, it was likely that the YAP transcriptional coactivator is upregulated in activated, cytotoxic CD8s. To test this hypothesis, SIINFEKL peptide was added to a heterogeneous immune cell suspension containing OTI+ CD8s, which were monitored for expression of YAP over time. This strong, Granzyme B- and IFNγ-inducing activation of CD8 cells upregulated the YAP message and protein expression by RT-qPCR and flow cytometry staining respectively (Figures 1D,E).

### CD8 Cells Lacking YAP Are More Potent Tumor Growth Suppressors

**Figure 2:**
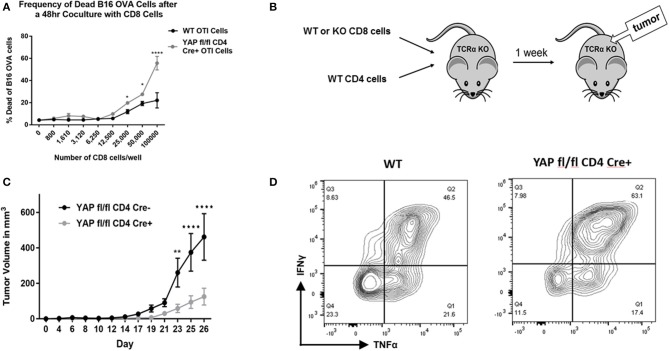
CD8 cells lacking YAP are more potent tumor growth suppressors. **(A)** Expanded enriched WT vs. YAP-deficient OTI CD8 cells were cocultured with B16-OVA cells in an *in vitro* killing assay. The frequency of live/dead B16-OVA cells was quantified using viability staining and flow cytometry. **(B)** Schematic explaining the adoptive transfer tumor experiments is depicted. **(C)** Expanded enriched WT or YAP-deficient OTI CD8 cells (along with WT CD4 cells) were adoptively transferred into TCRα KO animals. One week later, the recipients were challenged with subcutaneous B16-OVA tumors. The ensuing tumor growth kinetics are depicted. **(D)** Tumor-infiltrating CD8 cells were stimulated and analyzed for cytokine expression using flow cytometry. Data information: **(A,C)** represent mean ± SEM. In **(A,C)** ^*^P < 0.05; ^**^P < 0.01; ^****^P < 0.0001 by a two-way ANOVA test. *N* = 4/group in **(A)** and *N* = 5/group in C. Results in **(A,C,D)** are representative of two independent experiments.

Already having the YAP fl/fl CD4 Cre+ mouse model, we further crossed these animals to the OTI background in order to observe any effect YAP may have on the antigen-specific killing function of CD8s. CD4-driven Cre recombinase expressed during the CD4/CD8 double-positive stage of T cell development in the thymus at least partially deletes the YAP gene in CD8+ cells (Figure S3). Next, the antigen-specific *in vitro* killing assay was performed. Specifically, activated OTI+ CD8s from YAP sufficient and deficient mice were co-cultured with OVA-expressing B16F10 murine melanoma tumor cells. In this system, activated OTI+ cells are able to effectively kill the tumor cells *in vitro*. After 24 and 48 h of coculture, the frequency of killed vs live tumor cells was quantified using flow cytometry. The OTI+ CD8+ cells lacking YAP exhibited a superior ability to kill OVA-expressing tumor cells (Figure 2A). This suggested that YAP normally suppresses cytotoxicity of activated CD8 cells.

Next, it was examined whether YAP-deficient CD8+ cells contribute to the very strong anti-tumor immunity that YAP CD4 Cre mice exhibit using an adoptive transfer model. To this end, TCRα KO mice (lacking αβ T cells) were reconstituted with WT CD4+ cells and WT or YAP knockout (KO) CD8+ cells (Figure 2B). (YAP fl/fl OTI+ CD4 Cre– vs YAP fl/fl OTI+ CD4 Cre+ littermate animals were used in all experiments to reduce any effects of genetic mismatch.) One week later, the animals were challenged with a large dose (one million) of B16-OVA tumor cells, injected subcutaneously in the back region. Disease progression was monitored over time, and the animals were harvested 26 days after the tumor challenge. The escape of the tumors from killing by the OTI+ cells occurs due to the loss of OVA expression by the tumor cells, a normal immunoediting process in cancer. The mice that received YAP-deficient CD8+ cells controlled tumor escape longer than those receiving WT CD8s (Figure 2C). This result suggested that YAP reduces cytotoxicity of CD8+ cells. Flow cytometry analysis of tumor-infiltrating CD8s revealed that YAP-deficient cells produce more IFNγ and TNFα (Figure 2D).

### YAP KO CD8 Cells Exhibit Enhanced Cytotoxic Cytokine Production and Depressed Expression of Immunoregulatory Molecules

**Figure 3:**
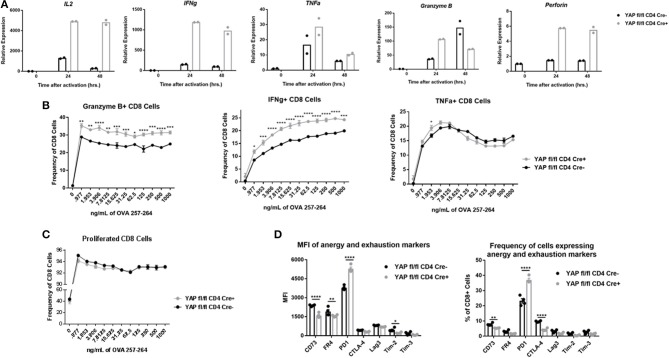
YAP KO CD8 cells exhibit enhanced cytotoxic cytokine production and depressed expression of immunoregulatory molecules. **(A)** mRNA expression of cytokines in activated (24 h) enriched OTI WT vs YAP-deficient CD8+ cells. **(B)** OTI spleen cells from WT and YAP-deficient animals were stimulated with IL2 and indicated concentration of SIINFEKL peptide. After incubation with monensin, the cells were tested for expression of Granzyme B, IFNγ, and TNFα via intracellular flow cytometry. **(C)** OTI spleen cells from WT and YAP-deficient animals were stained with Cell Trace Violet prior to stimulation for 72 h with SIINFEKL peptide and IL2. Proliferation was quantified via flow cytometry. **(D)** Spleen and LN homogenates from OTI+ YAP fl/fl CD4 Cre+ and CD4 Cre- mice were activated with SIINFEKL peptide in the presence of IL2 for 96 h. Surface expression of the indicated molecules was quantified using flow cytometry. Data information: **(B,C,D)** represent mean ± SEM. ^*^P < 0.05; ^**^P < 0.01; ^***^P < 0.001; ^****^P < 0.0001 by a two-way ANOVA test. *N* = 4/group in **(B–D)**. Results in **(B–D)** are representative of two independent experiments.

YAP fl/fl FoxP3 Cre+/+ CD4 Cre+ animals (YAP deleted in all T cells) have slightly enlarged lymph nodes and spleen than YAP fl/fl FoxP3 Cre+/+ CD4 Cre- animals, in which YAP is deleted only in Tregs (Figure S4). This observation further enforces the notion that YAP plays an anti-inflammatory role in non-Treg cells. The enhanced resistance of animals lacking YAP in CD8+ T cell subset to tumor progression was further elucidated with several *in vitro* observations. To begin, spleen and LN homogenates from OTI+ YAP fl/fl CD4 Cre- or CD4 Cre+ animals were activated with SIINFEKL peptide in the presence of IL2 before being enriched for the CD8 fraction at indicated times. The cytokine mRNA transcripts were generally in higher abundance in the YAP-deficient group as quantified by qRTPCR (Figure 3A). The expression of Granzyme B was lower in the KO group at 48 h, suggesting that YAP plays a suppressive role in early activation of CD8s. RNAseq analysis of sorted YAP-sufficient vs YAP-deficient OTI cells confirmed that deletion of YAP enhanced the cytokine signature, meanwhile the canonical Hippo pathway direct target genes were largely unaffected (Figure S5) (24–28). The expression of Granzyme B, IFNγ, and TNFα were also quantified using flow cytometry after the cells were incubated with monensin, a protein transport inhibitor. A greater frequency of YAP KO CD8+ cells expressed Granzyme B and IFNγ, but not TNFα, compared to WT CD8+ cells (Figure 3B). However, the cytokine production of CD4+ cells and the proliferation of either CD4+ or CD8+ cells (cells were stained with cell trace violet prior to stimulation) were not affected, enforcing the notion that potential effect of a small population of contaminating Tregs did not play a notable role in this system (Figure 3C and Figure S6). Finally, deletion of YAP in CD8 cells did not affect the expression of canonical YAP/TAZ target genes (Figure S7). This evidence suggests that YAP activity is unique in different cell types.

We also aimed to determine whether YAP had any long-term effect on the activation state of CD8 cells. After 4 days *in vitro* stimulation culture conditions, the OTI cells from the YAP KO group expressed less surface molecules associated with anergy and exhaustion compared to the WT group, with the exception of PD-1 (Figure 3D). This was particularly interesting, because PD-1 is oftentimes upregulated in highly-activated CD8 cells. These results further supported an anti-inflammatory role of YAP during activation of CD8 cells.

### YAP Is Relevant in Human CD8 Cells

**Figure 4:**
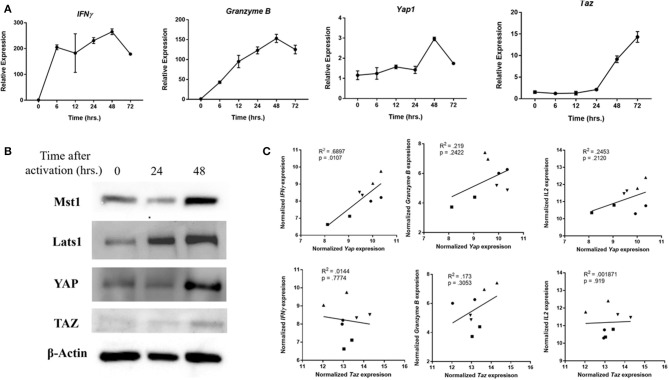
YAP expression is relevant in human cells. **(A)** Human CD8 cells were activated, and collected at indicated times. mRNA expression in these samples was quantified using RTqPCR. **(B)** Western Blot of human CD8 cells depicts upregulation of Lats and YAP at indicated times after cell stimulation. **(C)** Expression of *Yap* in PBMC-derived CD8s was correlated to that of *Granzyme B* and *IFN*γ using RT-qPCR. Each different symbol represents an individual donor. Data information: **(A)** represents mean ± SEM. *P* and *R*^2^ were calculated by Prism Software's linear regression test.

When human PBMC-derived CD8 cells were activated *in vitro*, expression of *Yap1* was upregulated with similar kinetics to that of *IFN*γ and *Granzyme B* (Figure 4A). This was verified using immunoblotting (Figure 4B). Other Hippo pathway members including Mst1, Lats1, and TAZ were also upregulated. Functionally, the expression of *Yap1* in human PBMC-derived CD8 cells correlated with the expression of *IFN*γ, *Granzyme B*, and *IL2* (Figure 4C). Although it appeared that TAZ was also upregulated during activation of human CD8s, its expression in peripheral blood CD8 T cells did not correlate with that of effector cytokines. Finally, single-cell melanoma TILs RNAseq data was used to investigate the activity of YAP and TAZ. CD8 cells that expressed *Yap1* had the most significantly upregulated IFNγ gene signature compared to those that did not (Figure S8). CD8 cells that expressed *Taz* had a completely different gene enrichment profile. Thus, it appears that YAP activity in tumor-infiltrating CD8 cells is uniquely linked to the pro-inflammatory IFNγ response.

## Discussion

Non-canonical roles of Hippo pathway components are rapidly being discovered in immune cells. While YAP is well known as an oncogene that promotes tumor progression, there is mounting evidence that YAP plays a considerable role in directing the suppression of anti-tumor immunity (13–17). Studies have demonstrated that YAP is upregulated in not only cancer cells, but also by the T regulatory (Treg) subset of CD4+ cells (13, 14). Activation of murine naïve CD8 cells in presence of antigen and IL-2 has also already been shown to increase transcription of the Hippo pathway components—including YAP—by Thaventhiran et al. (29). However, binding of CTLA-4 to CD80/86 between two activated CD8 cells caused activation of the Hippo signaling pathway that degraded YAP. In this report, authors showed that Hippo-mediated degradation of YAP was required for the CD8s to gain expression of Blimp-1 and the senescence-associated KLRG1, suggesting that these events promote terminal differentiation and limit clonal expansion of the CD8 cells. Nevertheless, it was unclear what function the upregulation of Hippo pathway components and YAP played in activated CD8 cells. One study reported that interferon regulator factor 3 (IRF3) promotes nuclear translocation and activity of YAP in response to antiviral signaling in gastric cell lines, and another that deletion of YAP and TAZ in cardiomyocytes caused severe IFNγ-driven pericardial inflammation and fibrosis (30, 31). However, such evidence falls short of associating the terminal Hippo pathway effectors YAP and TAZ to a functional role in CD8 cells, and the therapeutic potential for targeting these factors in the cancer setting remains incompletely explored.

YAP and TAZ transcriptional effectors of the Hippo signaling pathway are of most interest, but—in our experience—have been difficult to study due to their low expression or rapid degradation in immune cells. Our observations strongly suggest that T cell activation promotes YAP expression and function in CD8 cells. Although it is unclear exactly which molecular players are involved, YAP could be upregulated in response to cytoskeletal mechanotransduction after the engagement of the TCR and formation of the immunological synapse (32). Recently it was reported that YAP is rendered active through a glycoprotein that is involved in sensing of extracellular matrix cues (33). Given that engagement of the TCR with the peptide-MHC of antigen presenting cells results in rearrangements of the cell membrane and the cytoskeleton, it is very plausible that the immunological synapse promotes the activation-driven YAP expression in CD8 T cells.

In this report, we not only identified that YAP was upregulated by cytotoxic CD8 cells but also explored its immunosuppressive role in these cells. We found that YAP was tightly linked to IFNγ response in CD8 T cells, especially in activated cytotoxic cells usually found in the tumor microenvironment. Our observations suggest that YAP becomes expressed in activated CD8 cells, where it plays an immunosuppressive role to help prevent excessive activation of and killing by these T cells. While deletion of YAP in CD8 cells enhanced cytotoxicity and expression of inflammatory cytokines, it did not affect the canonical cell proliferation or apoptosis pathways this transcriptional effector normally acts on in tumor cells. This was unexpected because YAP is best known for binding to the TEAD transcription factors that canonically regulate differentiation, proliferation, and apoptosis (34). More work is necessary to fully elucidate which transcription factor(s) YAP interacts with to exert this effect in CD8 cells. New evidence is emerging that YAP can associate with non-TEADs such as Smad2/3, but almost nothing is known about YAP's binding partners in immune cells (35). Nevertheless, our findings are significant because they support and help to explain efficacy of YAP inhibitors in the treatment of cancer, as YAP appears to not only play a pro-oncogenic role in tumor cells but also promotes suppression of both CD4 and CD8 T cells.

## Materials and Methods

### Mice

C57BL/6 YAP fl/fl mice were generously donated by Dr. Duojia Pan, TCRα KO mice by Dr. Hong Yu, and OTI mice by Dr. Chirag Patel and Dr. Jonathan Powell. C57BL/6 CD4-cre and Foxp3-YFP-Cre transgenic mice were purchased from the Jackson Laboratory. All animal experiments were performed under protocols approved by the Johns Hopkins University Institutional Animal Care and Use Committee (IACUC). Primarily, 6-12 week-old female mice were used for animal experiments.

### Tumor Models

Murine B16F10 melanoma cells, MC38 colon adenocarcinoma, and EL4 thymoma cell lines were purchased from the American Type Culture Collection (ATCC) and kept as frozen stock in 2015. These cell lines have not been authenticated by the laboratory. Cells were cultured as described by the company.

The back region of female animals (~8-weeks old) was shaved one day prior to subcutaneous (s.c.) injection if the indicated cell line in 100ul of PBS immediately after the cells were washed with ice-cold PBS. 40,000 of B16F10, 250,000 MC38, or 200,000 EL4 cells were injected per mouse, unless otherwise indicated. Tumor progression was quantified using the formula V = (L^*^W^2^)/2, where V is volume, L is maximum length, and W is width perpendicular to the length. Excised tumors were weighed using a balance.

### Cell Isolation

Generally, spleen and lymph nodes from YAP fl/fl FoxP3 YFP Cre+ CD4 Cre- or CD4 Cre+ animals were harvested into 100 μm cell strainers (Falcon) in 5% FBS RPMI 1640. The organs were ground through the cell strainers using sterile 3 mL syringes. Red blood cells were lysed using ACK lysis buffer (Quality Biological). When pure CD8s were needed, the cells were enriched using Miltenyi Biotec's Mouse CD8 Isolation Kit per manufacturer's protocol. Cells were spun down at 400 g for 5 min.

### Human Blood

Human blood leukopacks were from the Johns Hopkins School of Medicine Division of Gastroenterology and Hepatology. De-identified peripheral blood mononuclear cells (PBMCs) were isolated from whole blood using Ficoll-Paque PLUS (GE Healthcare) density centrifugation. Single-use aliquots of cells (frozen in 10% DMSO, 40% FBS RPMI 1640) were stored in liquid nitrogen. CD8 T cells were enriched using MojoSort Human CD8 T Cell Isolation Kit (BioLegend) using manufacturer's protocol.

### Cell Sorting

Homogenized spleen, lymph node, and/or tumor cells were stained with defining antibodies as indicated in FACS buffer. Cells were sorted using FACS Aria Instrument with the assistance of the Sidney Kimmel Comprehensive Cancer Center Flow Cytometry Core staff into complete RPMI medium (5% FBS), before being spun down to be stored in TRIzol.

### Flow Cytometry

Cell Trace Violet (Invitrogen) was used to track cell proliferation according to manufacturer's protocol. Cells were either stained directly or incubated in 1x Monesin in 5% FBS RPMI 1640 for 4 h before detection of cytokines. Cells were stained with a viability dye for 20 min at room temperature in PBS, with fluorophore-conjugated antibodies against surface markers for 15 min at room temperature in FACS buffer, and with fluorophore conjugated antibodies against intracellular markers for 30 min at room temperature in permeabilization wash buffer. Both BD Cytofix and ThermoFisher Transcription Factor fixation kits were used, depending on the antibodies. The list of antibodies and primers used in the study can be found in the Supplementary Data Sheet 2.

### Statistical Analysis

All statistical analyses were done using Prism 7 Software. Unpaired two-tailed student's *t*-test was used to compare means between two groups, and a two-way analysis of variance (ANOVA) was used for determine statistical significance of data that had more than two groups. Values are presented as means ± SEM where appropriate. ^*^p < 0.05, ^**^p < 0.01, ^***^p < 0.001, and ^****^ *p* < 0.0001.

### RT-qPCR

Cells were lysed using TRIzol (Invitrogen) reagent. After undergoing phase-separation with chloroform, RNA was purified using Direct-zol RNA Purification Kit (Zymo Research) according to manufacturer's protocol. Equal amounts of total RNA (as measured by NanoDrop Spectrophotometer) were converted to cDNA using SuperScript III Reverse Transcription Kit (Invitrogen) as described by the manufacturer. Gene expression was quantified using SYBR Green RT-qPCR Master Mix (ThermoFisher Scientific) with gene specific primers. Primer sequences were either obtained from the Harvard Primer Bank or designed using NCBI Primer Bast Software. All primers were validated with dose-dependent amplification using RT-qPCR, with melt curves, and with DNA gel electrophoresis.

### OTI Activation

Spleen and lymph node cell suspension was activated with up to 1,000 ng/ml SIINFEKL peptide (purchased from the Johns Hopkins School of Medicine Synthesis and Sequencing Facility) in 5% FBS RPMI 1640 in the presence of 2ng/mL recombinant IL2 (PeproTech). Cells were split daily with media containing fresh IL2.

### Immunoblotting

Cell suspensions were spun down, washed with PBS, and lysed with RIPA buffer. After at least one freeze-thaw cycle, the lysate was centrifuged. The protein in the soluble fraction was then quantified using Pierce BCA Protein Assay Kit according to manufacturer's protocols. Standard Western Blotting procedures were subsequently used using BioRad's reagents, equipment, and standard protocols. All blots were incubated with primary antibodies in PBS overnight at 4°C.

### Analysis of scRNA-Seq Datasets

The published expression data of cells passing quality control filters in human melanoma (GSE72056) were downloaded, and then re-processed using the Seurat analysis pipeline (36). CD8+ T cells were extracted for downstream gene set analysis. Differential expression between Yap+ CD8+ and Yap- CD8+ was compared within each pathway using the hallmark pathway fgsea package.

## Data Availability Statement

All datasets generated for this study are included in the article/Supplementary Material.

## Ethics Statement

The animal study was reviewed and approved by Johns Hopkins University IACUC.

## Author Contributions

FP and DP designed, supervised the experiments, and edited the manuscript. AL performed almost all experiments, collected and analyzed the data, and wrote the manuscript. LC performed bio-computational analysis and assisted in data acquisition.

### Conflict of Interest

The authors declare that the research was conducted in the absence of any commercial or financial relationships that could be construed as a potential conflict of interest.
