# Supplementary material for: YAP Attenuates CD8 T Cell-Mediated Anti-tumor Response
Source: Front Immunol. 2020 Apr 8;11:580. doi: 10.3389/fimmu.2020.00580 (PMC7158852; doi:10.3389/fimmu.2020.00580)
Supplement: Supplementary file 1 [file Data_Sheet_1.PDF]

Supplementary Figure 1

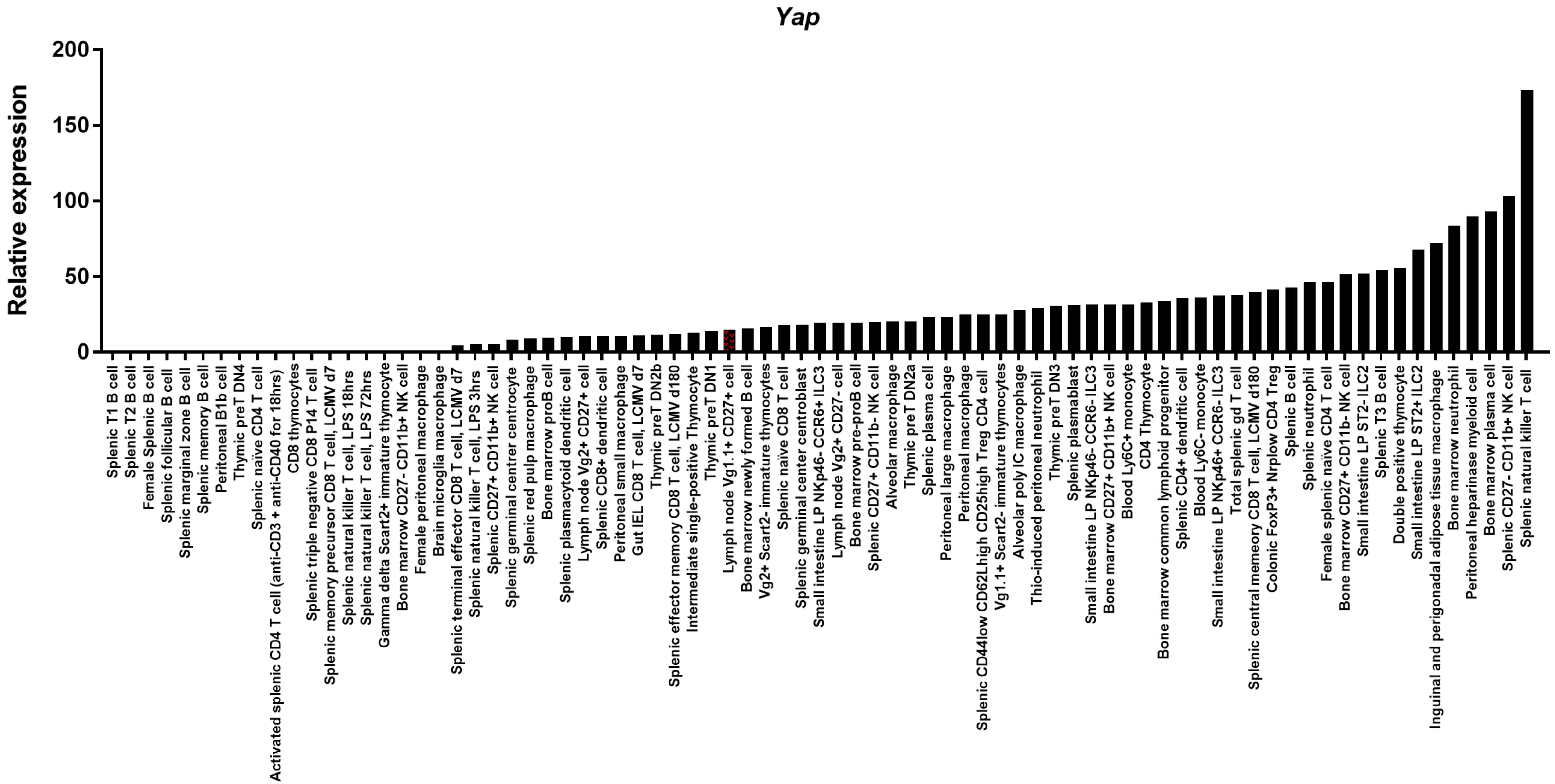

Supplementary Figure 1

RNAseq data from the Immgen Skyline database compares *Yap1* expression in various murine immune cell subsets. Relative expression values from the database were replotted using Prism software.

Supplementary Figure 2

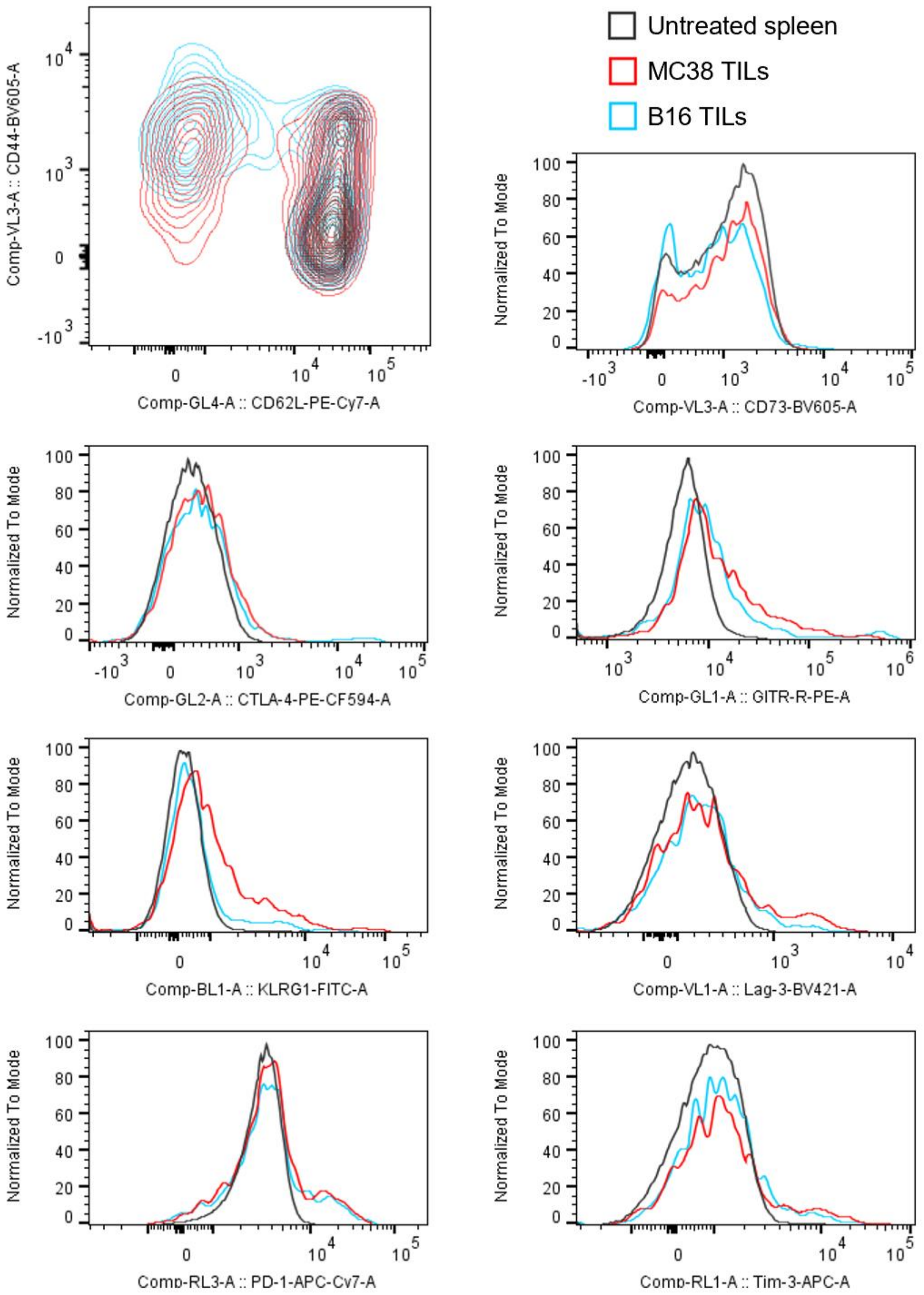

Supplementary Figure 2

Immune cell suspensions from indicated sources were stained for various activation and exhaustion markers, and examined by flow cytometry. Shown plots are of live CD3+ CD8+ cells.

Supplementary Figure 3

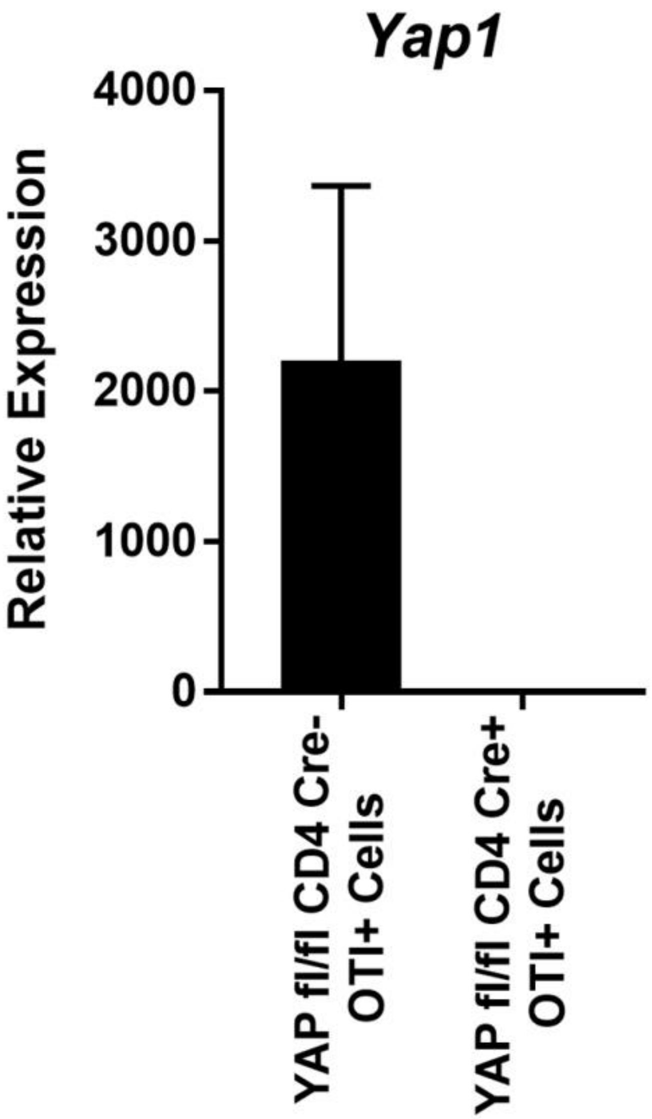

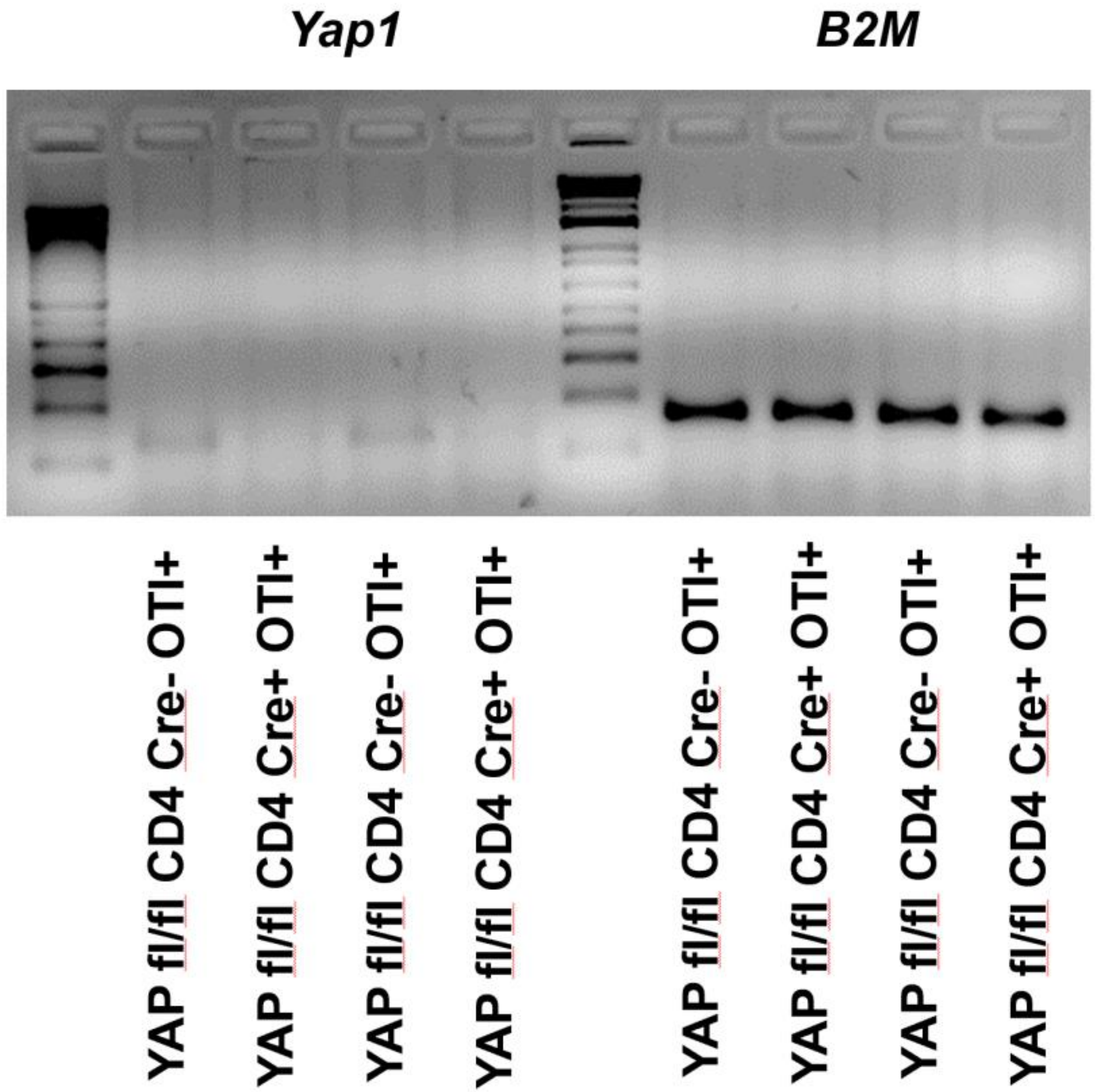

Supplementary Figure 3

A. *Yap1* mRNA differential expression in activated enriched OTI+ CD8+ cells, normalized to *B2M*. *Yap1* primer was designed to span the deletion region (defined by loxP sites). **B.** DNA gel depicting *Yap1* and *B2M* RT-qPCR products.

Supplementary Figure 4

YAP fl/fl Foxp3 Cre+/+
OT1+CD4 Cre-

YAP fl/fl Foxp3 Cre+/+
OT1+CD4 Cre+

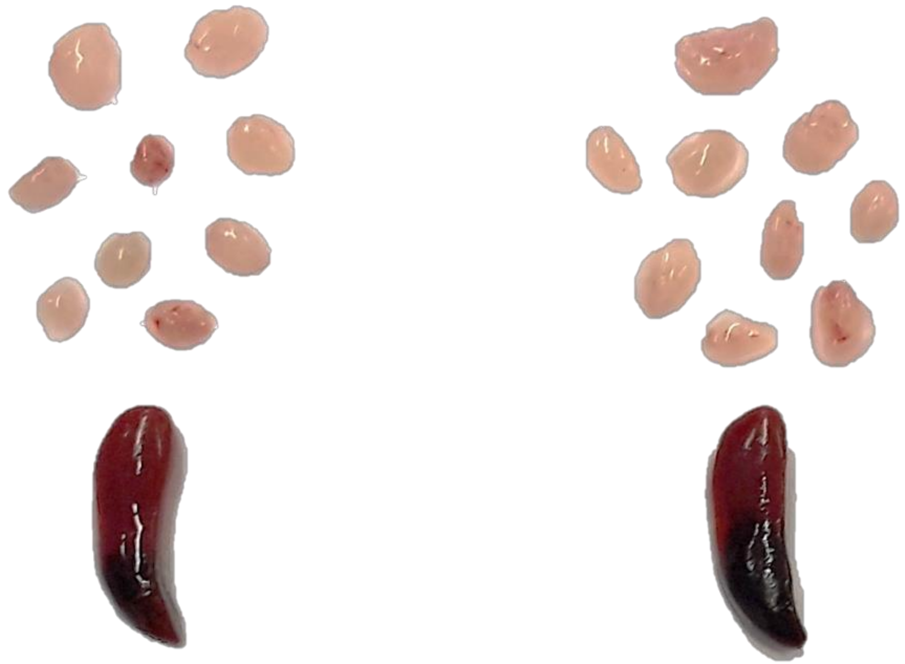

Supplementary Figure 4

Excised spleen and lymph nodes of indicated animals are depicted.

Supplementary Figure 5

A

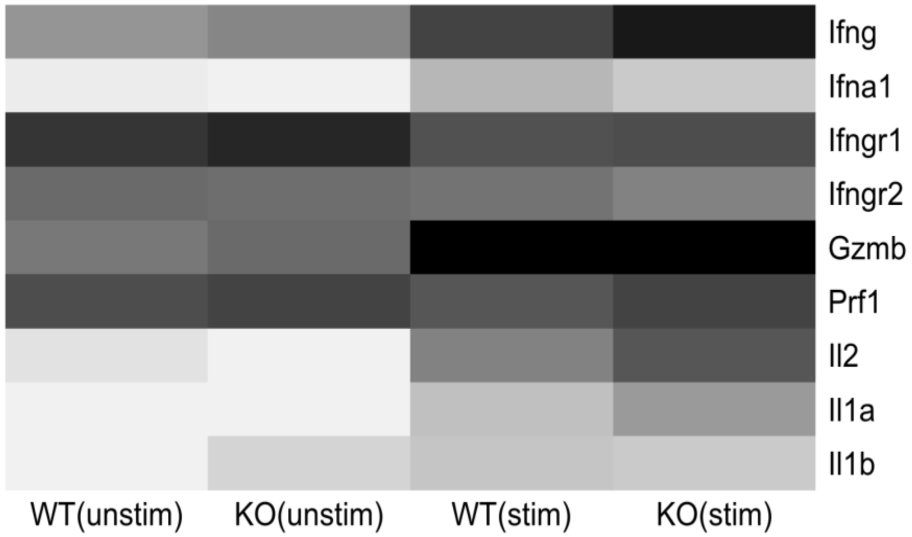

B

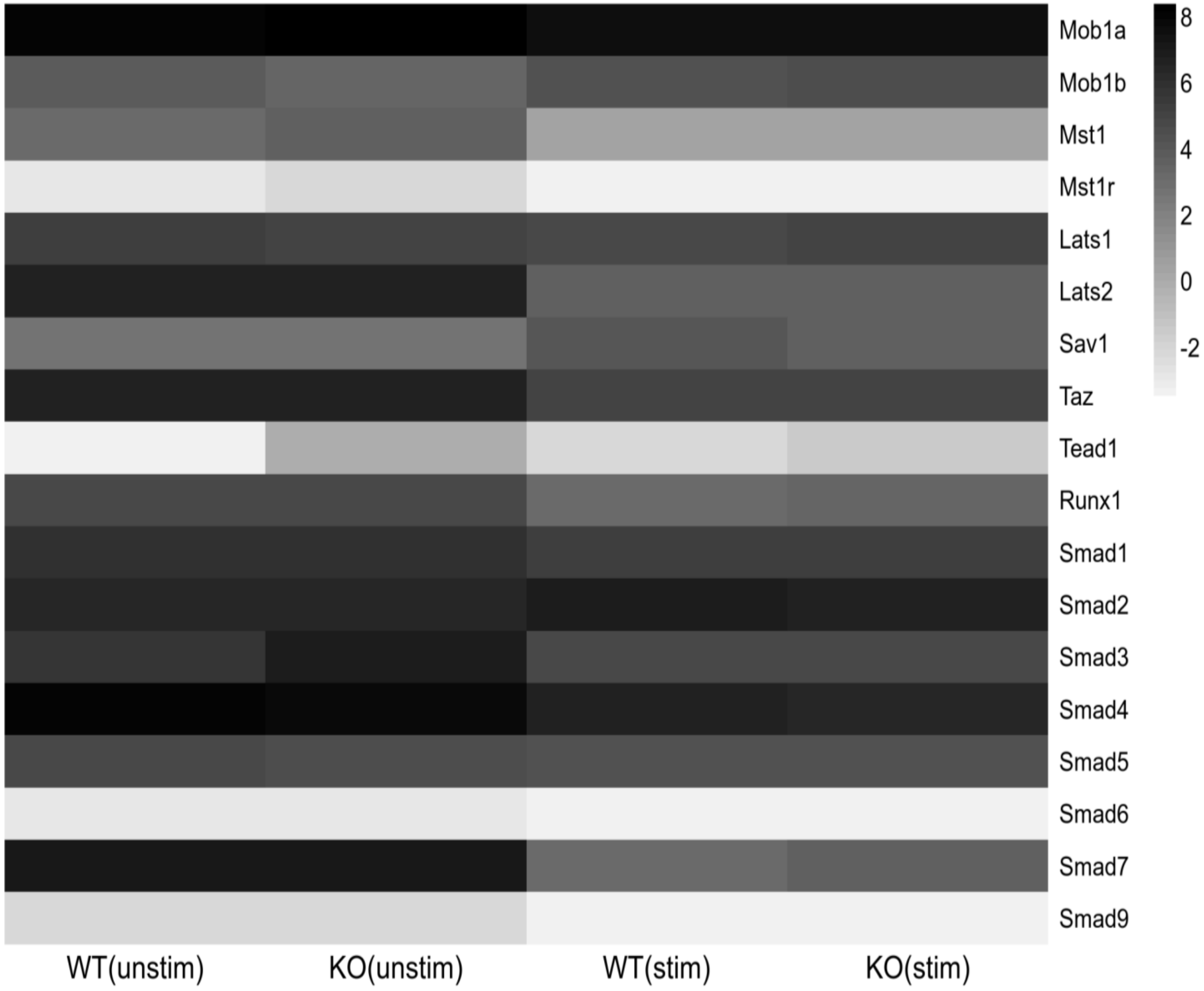

Supplementary Figure 5

A. Heatmap of RNAseq CD8 T cell cytokine gene expression signature (logarithmic scale) in described groups. OTI cells from YAP fl/fl CD4 Cre- FoxP3 Cre+/+ and YAP fl/fl CD4 Cre+ FoxP3 Cre+/+ mice were sorted before and after being activated with SIINFEKL in the presence of IL-2 for 48 hours. **B.** Heatmap of RNAseq Hippo pathway gene expression signature in described groups.

Supplementary Figure 6

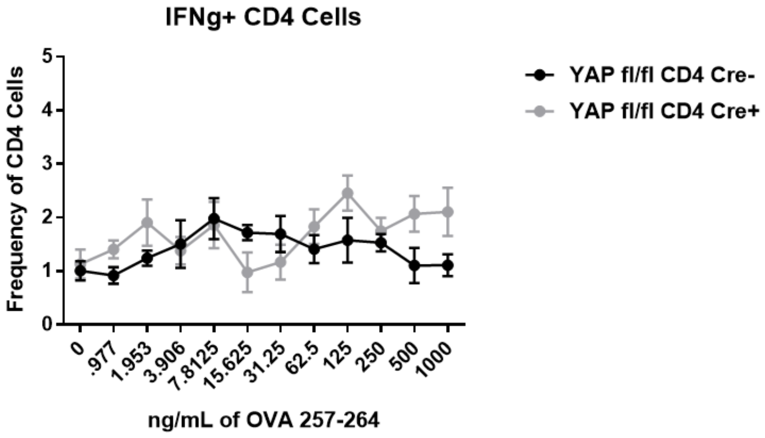

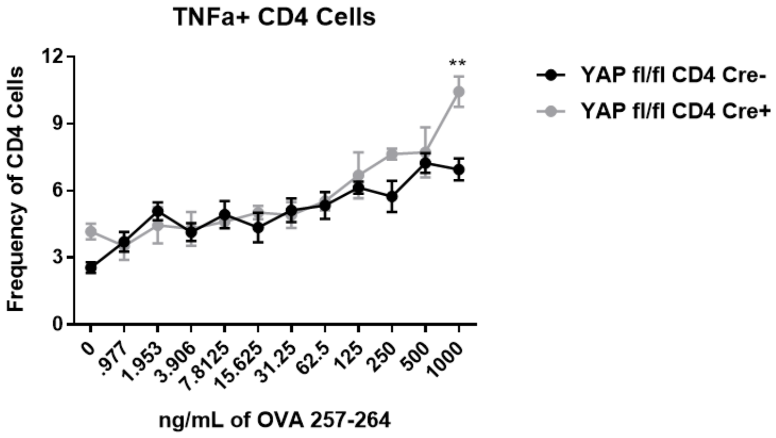

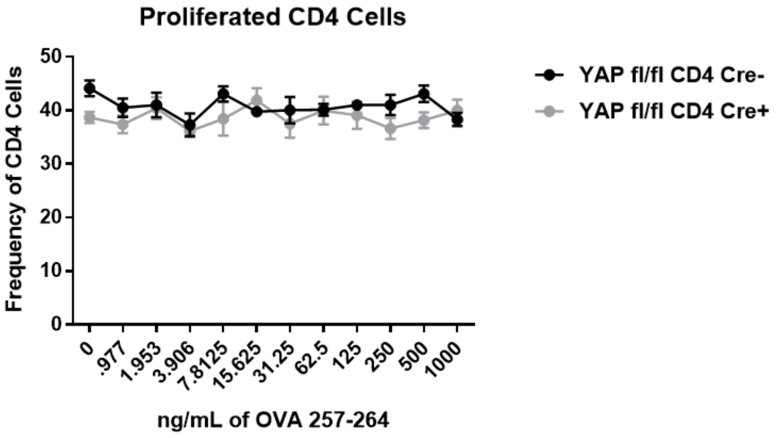

Supplementary Figure 6

IFNγ and TNFα production and proliferation by SIIFEKL-activated OTI+ splenocytes in the presence of IL-2 was quantified using flow cytometry.

Supplementary Figure 7

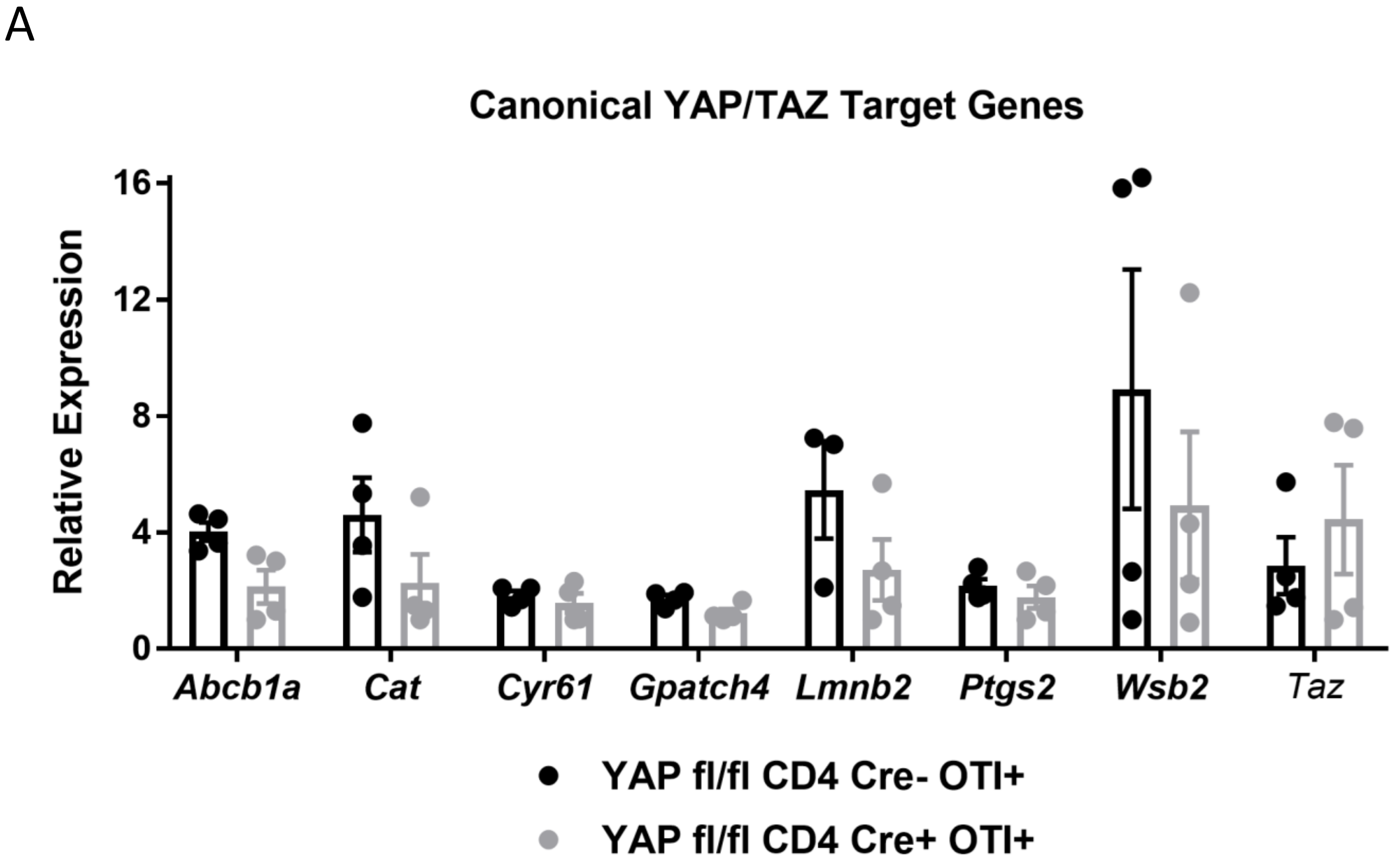

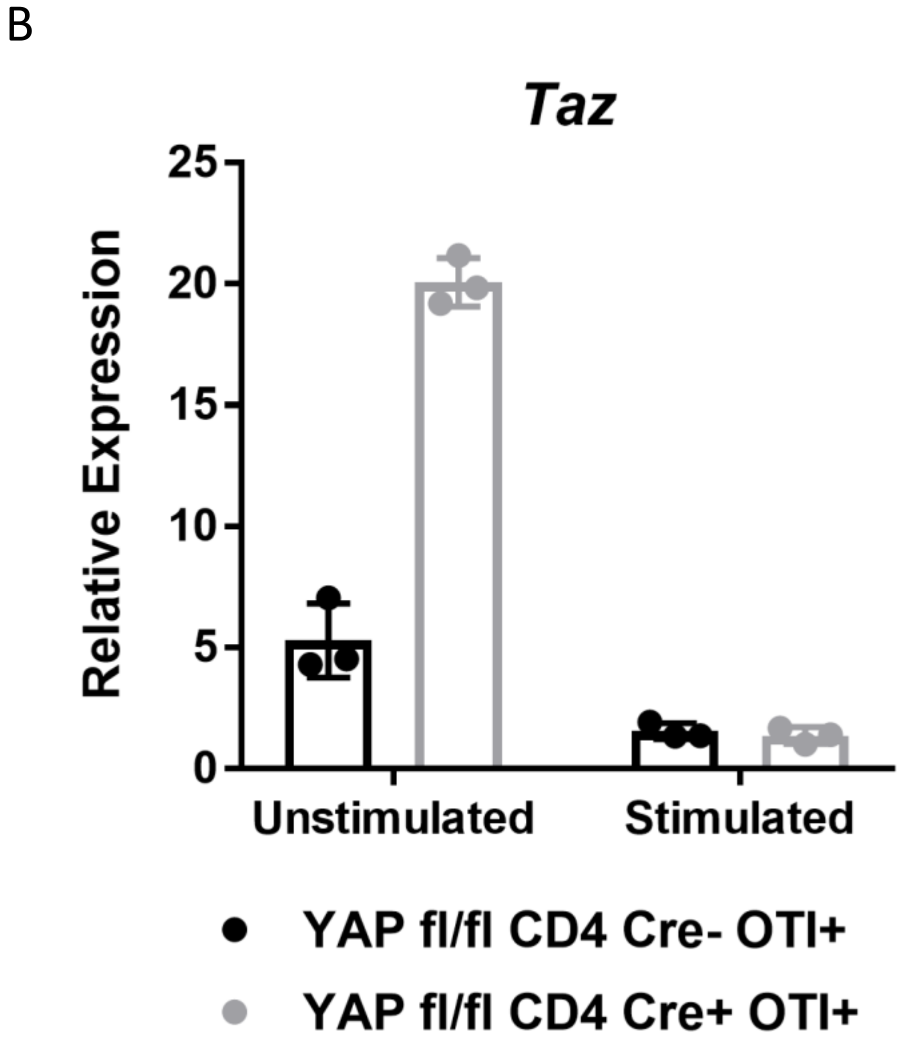

Supplementary Figure 7

A. Differential expression of canonical Hippo pathway target genes in enriched OTI+ CD8+ cells activated for 48 hours by SIINFEKL-loaded antigen presenting cells in the presence of IL-2, and normalized using *B2M*. **B**. Differential gene expression of *Taz* in YAP-sufficient vs YAP-deficient animals. Enriched cells were stimulated with SIINFEKL in the prece of IL-2 for four days.

Supplementary Figure 8

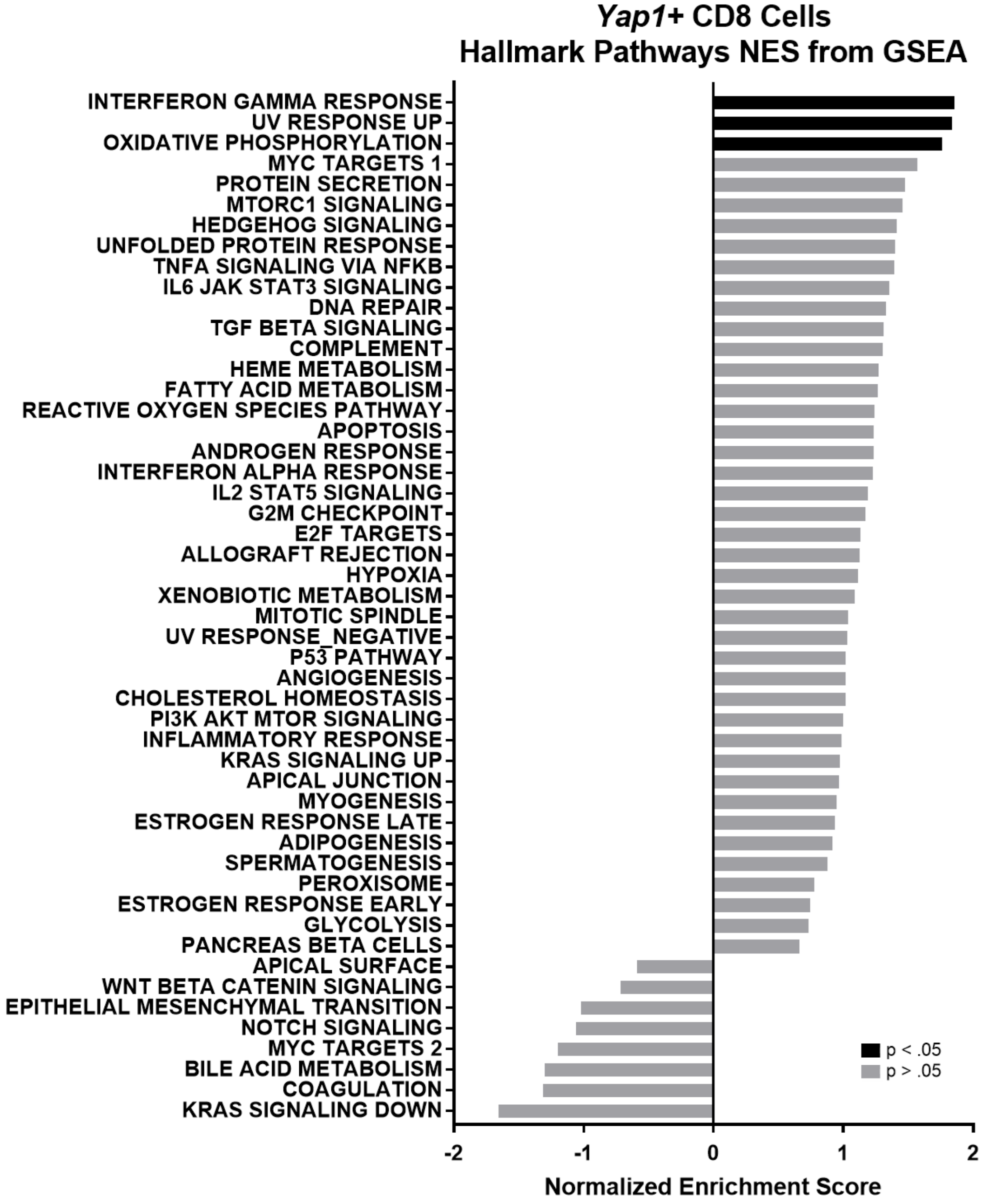

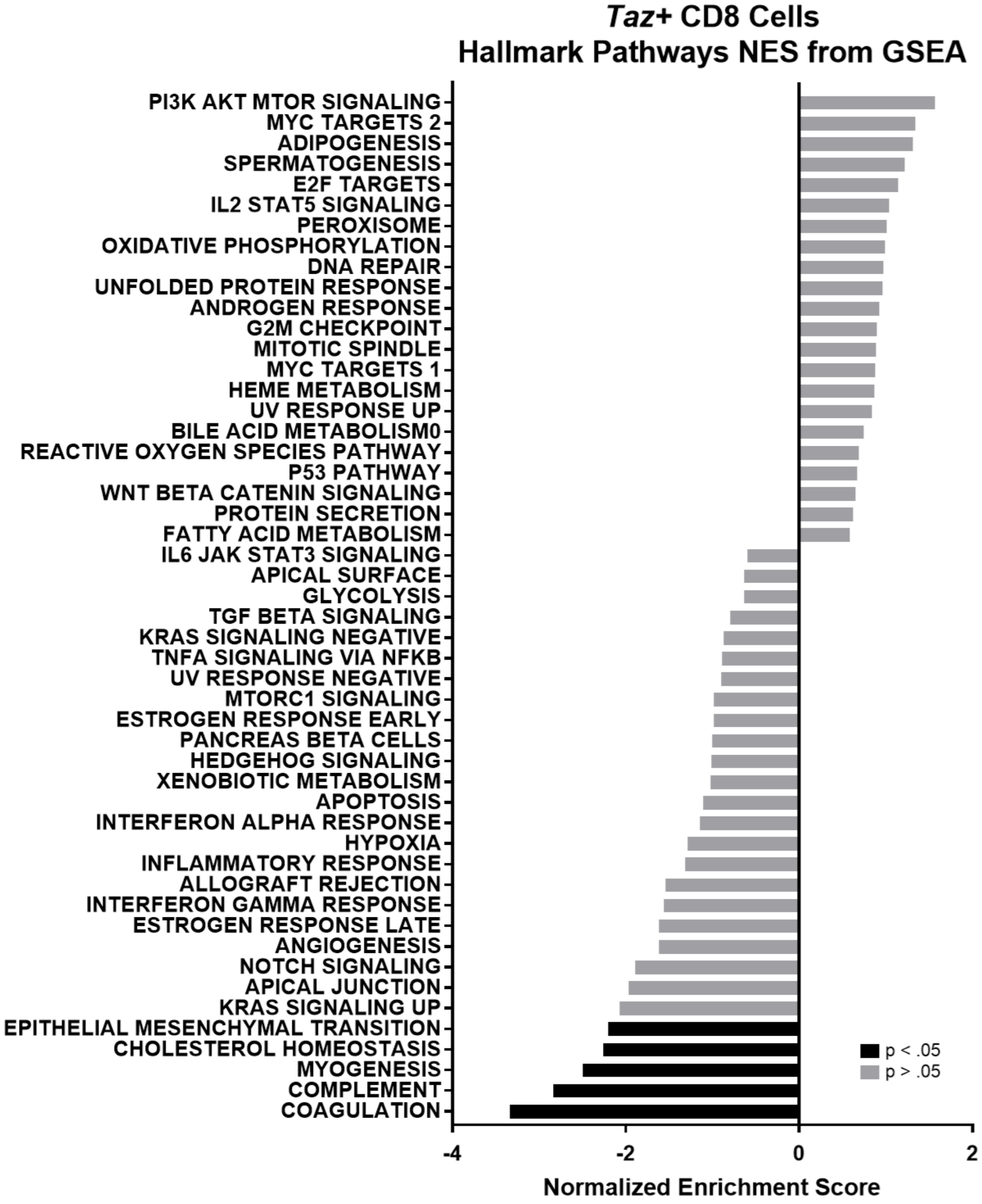

Supplementary Figure 8

Human melanoma TIL single cell RNAseq data was analyzed for indicated transcriptional signatures. CD8+ cells were segregated into *Yap1*+ vs *Yap*- and *Taz*+ vs *Taz*- groups. Enrichment scores represent up- or down-regulated signatures in cells that expressed the transcriptional coactivators compared to those that did not.
