# Supplementary material for: YAP Attenuates CD8 T Cell-Mediated Anti-tumor Response
Source: Front Immunol. 2020 Apr 8;11:580. doi: 10.3389/fimmu.2020.00580 (PMC7158852; doi:10.3389/fimmu.2020.00580)
Supplement: Supplementary file 2 [file Data_Sheet_2.pdf]

Flow cytometry antibodies - Mouse

Marker	***Clone***	***Manufacturer***
CD3	17A2	BioLegend
CD4	GK1.5	BioLegend
CD8	53-6.7	BioLegend
CD11b	M1/70	BioLegend
CD19	6D5	BioLegend
CD25	3C7	BioLegend
CD73	TY/11.8	BioLegend
Granzyme B	REA226	Miltenyi Biotec
Perforin	S16009A	BioLegend
IL2	JES6-5H4	BioLegend
IFNγ	XMG1.2	BioLegend
TNFα	MP6-XT22	BioLegend
FoxP3	FJK-16s	ThermoFisher Scientific
FR4	12A5	BioLegend
PD-1	29F.1A12	BioLegend
Lag3	C9B7W	BioLegend
CTLA-4	UC10-4B9	BioLegend
Tim2	F37-2C4	BioLegend
Tim3	RMT3-23	BioLegend
YAP	D8H1X	Cell Signaling Technology

Flow cytometry antibodies - Human

Marker	***Clone***	***Manufacturer***
CD3	SK7	BioLegend
CD4	SK3	BioLegend
CD8	SK1	BioLegend
Granzyme B	REA226	Miltenyi Biotec

IL2	MQ1-17H12	BioLegend
IFNγ	4S.B3	BioLegend

Primers used in this study - Mouse

Gene	*Forward primer (5' to 3')*	*Reverse primer (5' to 3')*
Yap1	TACTGATGCAGGTACTGCGG	TCAGGGATCTCAAAGGAGGAC
Taz	GAAGGTGATGAATCAGCCTCTG	GTTCTGAGTCGGGTGGTTCTG
B2M	CTCGGTGACCCTGGTCTTTC	GGATTTCAATGTGAGGCGGG
IL2	TGAGCAGGATGGAGAATTACAGG	GTCCAAGTTCATCTTCTAGGCAC
Granzyme B	CCACTCTCGACCCTACATGG	GGCCCCCAAAGTGACATTTATT
Perforin	CAAGGTAGCCAATTTTGCAGC	GTACATGCGACACTCTACTGTG
Abcb1a	CAGCAGTCAGTGTGCTTACAA	ATGGCTCTTTTATCGGCCTCA
Cat	AGCGACCAGATGAAGCAGTG	TCCGCTCTCTGTCAAAGTGTG
Cyr61	CTGCGCTAAACAACTCAACGA	GCAGATCCCTTTCAGAGCGG
Gpatch4	AGAGGCAGAAAGCCGAGAGA	CAGATTGCTCTGTGCTCAGTT
Lmnb2	TGGCATCAAGACCCTGTACGA	TCAGCCTCACTCCGGTGAAA
Ptgs2	TGAGCAACTATTCCAAACCAGC	GCACGTAGTCTTCGATCACTATC
Wsb2	CTTCTCGCCAGACGGTTCC	GGTGACGTGCCCAGAGTTTC

Primers used in this study - Human

Gene	*Forward primer (5' to 3')*	*Reverse primer (5' to 3')*
Yap1	TAGCCCTGCGTAGCCAGTTA	TCATGCTTAGTCCACTGTCTGT
Taz	TCCCAGCCAAATCTCGTGATG	AGCGCATTGGGCATACTCAT
RPL13A	GCCATCGTGGCTAAACAGGTA	GTTGGTGTTCATCCGCTTGC

Western blot antibodies

YAP	D8H1X	Cell Signaling Technology
TAZ	V386	Cell Signaling Technology

LATS1	9153	Cell Signaling Technology
MST1	D8B9Q	Cell Signaling Technology
